# The Impact of Salts on the Ice Recrystallization Inhibition Activity of Antifreeze (Glyco)Proteins

**DOI:** 10.3390/biom9080347

**Published:** 2019-08-06

**Authors:** Romà Surís-Valls, Ilja K. Voets

**Affiliations:** Laboratory of Self-Organizing Soft Matter, Laboratory of Macro-Organic Chemistry, Department of Chemical Engineering and Chemistry & Institute for Complex Molecular Systems, Eindhoven University of Technology, Post Office Box 513, 5600 MD Eindhoven, The Netherlands

**Keywords:** antifreeze proteins, ice recrystallization inhibition, hofmeister series

## Abstract

Antifreeze (glyco)proteins (AF(G)Ps) have received increasing attention as potential cryopreservation agents since their discovery in the 1970s. While cryopreservation strategies for specific cells (such as red blood cells) are successful and widely implemented, preservation of other cell types, tissues and whole organs remains challenging. This is due to the multifactorial nature of the freeze-thaw damage, the complexity of preserving biological matter and the (country-to-country) variability of the employed procedures and regulations. AF(G)Ps are well-known for their ability to modulate ice crystal growth morphology and ice recrystallization inhibition (IRI), both of which are considered key contributors to freeze-thaw damage. To date, however, the impact of AF(G)Ps on cell survival remains at best partially understood as conflicting results on the benefits or disadvantages of including AF(G)P in cryopreservation strategies remain unelucidated. We hypothesize that variability in the additives in the cryopreservation media contributes to the observed discrepancies. To critically examine this idea, we monitored the inhibition of ice recrystallization by AF(G)P in the presence of various salts using a quantitative analysis of optical microscopy images via the Lifshitz-Slyozov-Wagner (LSW) theory for Oswald ripening. We found that the addition of salts, which are used in culture and cryopreservation media, enhances the IRI activity of AF(G)Ps, and that the magnitude of the enhancement was in line with the Hofmeister series. The size of ice crystals grown in AFGP_1–5_ and type III AFP samples containing chloride, phosphate and citrate ions were statistically smaller after 90 min of incubation than crystals grown in the absence of these salts. The ice recrystallization rates (*k*_d_) of AFGP_1–5_ and type III AFP samples prepared at a fixed overall ionic strength of 100 mM progressively decreased following the Hofmeister series for anions. Our results demonstrate that the performance of AF(G)Ps is significantly influenced by additives present in common cryopreservation media. It is thus important to conduct excipient compatibility experiments to identify potential incompatibilities between additives and AF(G)Ps in cryopreservation formulations.

## 1. Introduction

While cryopreservation strategies for specific cells (such as red blood cells) are successful and widely implemented, preservation of other cell types, tissues and whole organs remains challenging. This is due to the multifactorial nature of freeze-thaw damage, the complexity of preserving biological matter and the (country-to-country) variability of the employed procedures and regulations. In many cases, cellular damage to cryopreserved cells, tissues and organs is induced by ice recrystallization during thawing [1,2]. Small molecules such as sugars, salts, alcohols and other small molecules are often used to mitigate these effects in a colligative fashion [2,3]. The disadvantage of this approach is the high concentration required to elicit a significant effect. Interestingly, antifreeze (glyco)proteins (AF(G)Ps) inhibit ice recrystallization at far lower concentrations, as they act in a non-colligative manner [4,5]. AF(G)Ps have thus received widespread attention as potential cryopreservation agents [6], yet with limited success in practice. Some studies have shown how exposure to AF(G)Ps results in loss of cell survival after freeze-thawing steps in cryopreservation protocols, but others have reported greater cell survival after using the same proteins at comparable concentrations [7,8,9,10]. We hypothesize that such discrepancies in literature are related to different additives in the cryopreservation media.

At the end of the 19th century, Franz Hofmeister observed and quantified egg white protein precipitation triggered by the addition of different salts containing the same cation. Hofmeister’s original work pointed towards ion specificity in the precipitation of proteins, where anions had a more pronounced effect than cations [11]. Direct ion-macromolecule interactions are likely responsible for most aspects of this singularity. Since Hofmeister’s seminal work, ion-specific effects in line with the so-called Hofmeister series have been observed throughout biology in enzyme activities, protein stability and protein–protein interactions among others [12,13,14]. Interestingly, the thermal hysteresis (TH) activity exhibited by AF(G)Ps—quantified as the magnitude of the gap between the melting and freezing temperatures of an AF(G)P solution—was found to increase in the presence of various salts in accordance with the Hofmeister series [15,16,17,18]. Kristiansen and collaborators suggested that this is due to enhanced ice adsorption driven by a salt-induced reduction in AFP solubility in the solution surrounding the ice crystal [17]. While this TH enhancement by salts is well documented, their role in ice recrystallization inhibition (IRI) activity remains unresolved [19]. It is likely that salts also significantly alter IRI, as both IRI and TH activity originate from the ability of AF(G)P’s to adhere to ice [5]. In this report we study the effect of salts from the Hofmeister series on the IRI activity of AF(G)Ps using the so-called ‘sucrose sandwich assay’ of IRI activity, which maintains a constant and low-ice volume fraction and allows the quantification of ice growth rate constants (*k*_d_) [20,21,22]. We found that salts common in cryopreservation media enhanced the IRI activity of AF(G)Ps and that the magnitude of the enhancement was in line with the Hofmeister series. These findings suggest that systematic experiments of the compatibility between excipients and AF(G)Ps in cryopreservation formulations may shed further light on observed variabilities in the performance of AF(G)Ps as cryoprotectants.

## 2. Materials and Methods

### 2.1. Materials

Salt stock solutions of 1 M were prepared by dissolving the desired amount of salt in ultrapure water. Sodium nitrate (NaNO_3_) > 99%, sodium phosphate (NaHPO_4_) > 89%, sodium citrate (Na_3_C_6_H_7_·2H_2_O), disodium tetraborate (Na_2_B_4_O_7_·10H_2_O) > 99.5% and sodium chloride (NaCl) > 99% (Acros Organics) were purchased from Fischer Scientific. AFGP_1–5_ and recombinant type III AFP QAE HPLC12 samples were prepared by mixing the protein with the different salted buffers in the right amounts to reach fixed protein concentrations of 5 nM (AFGP_1–5_) and 500 nM (rQAE), which are the IRI activity inflection points in the absence of added salts according to literature [5].

### 2.2. Ice Recrystallization Inhibition (IRI) Assay

To test the effect of specific ion interactions on the IRI activity of AF(G)Ps that bind the primary prism plane, we selected the sucrose sandwich assay. Classical quantitative methods for studying IRI activity, such as the splat assay and the capillary method, are not sensitive enough to detect small differences in IRI. The liquid volume fraction is too low and the ice volume fraction too high to reliably avoid false positives (i.e., to avoid qualifying (macro) molecules without ice affinity as inhibitors of ice recrystallization). In a sucrose sandwich assay, the liquid volume fraction is increased by addition of the innocuous solute sucrose, allowing molecules without ice affinity to freely defuse. IRI experiments are performed at a fixed concentration of 5 nM (AFGP_1–5_) and 500 nM (rQAE). We prepared these by sandwiching 2 µL of sample between two cover slides, which was rapidly frozen (20 °C/min) to −40 °C in a Linkam LTS420 stage attached to a Nikon ECLIPSE Ci-Pol Optical Microscope (Figures S1 and S2). The samples were then annealed at −7 °C for 90 min, while microphotographs were taken every 2 min to follow the ice growth over time. All samples showed well-defined grain boundaries and frozen fractions lower than 0.3, which made them suitable for the study of migratory recrystallization and minimized ice crystal accretion (Figure S3).

### 2.3. Statistical Analysis

The statistical analysis was performed in OriginPro 2015 (OriginLabs). Normal distributions of ice crystal radii were assessed by Shapiro–Wilk tests to properly analyze the statistical differences between data sets. The non-parametric Mann–Whitney–Wilcoxon test was used to identify statistical differences between the median values for the ice crystal radii at the end point of the experiment; p-values lower than 0.05 indicated statistical differences between compared sets.

## 3. Results and Discussion

Aiming to elucidate the effect of various salts commonly used in cryopreservation media, we compared the ice recrystallization inhibition (IRI) activity of antifreeze (glyco)proteins in the presence of sodium salts from the Hofmeister series for anions at a fixed overall ionic strength of 100 mM. We selected two widely studied fish antifreeze proteins, which varied three orders of magnitude in IRI activity in 30 wt% sucrose solutions. These are the globular fish type III antifreeze protein QAE HPLC12 (rQAE) from the ocean pout, and the glycosylated polypeptide AFGP_1–5_ from Antarctic tooth fish. We analyzed the impact of sodium nitrate, sodium chloride, sodium borate, sodium phosphate and sodium citrate on their IRI activity by optical microscopy using the so-called sucrose sandwich assay, which allowed us to determine and monitor ice crystal grain size and ice growth rates over time.

**Figure 1:**
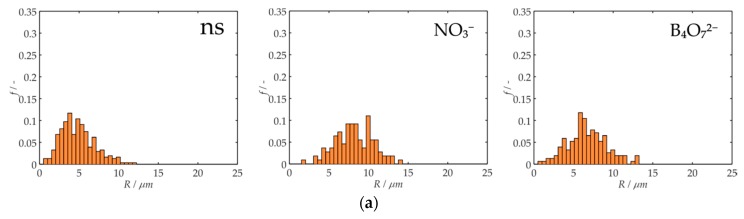

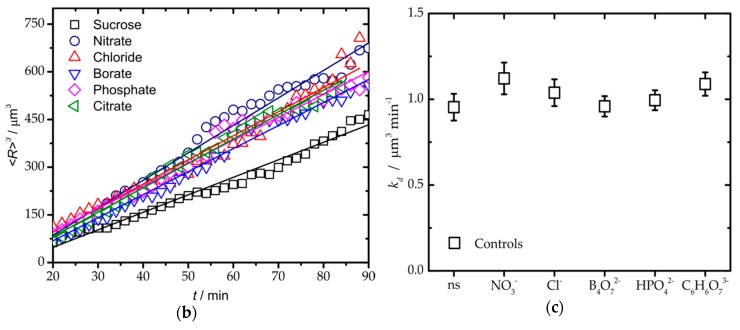
Analysis of the results obtained for reference solutions of the different salts studied. (**a**) Ice crystal radii distributions for a sample containing MilliQ water and 30 wt% sucrose (ns); 100 mM total ionic strength of sodium nitrate and 30 wt% sucrose (NO_3_^−^); and 100 mM total ionic strength of disodium tetraborate and 30 wt% sucrose (B_4_O_7_^2−^). (**b**) Samples containing 500 nM rQAE in 30 wt% sucrose (black), sodium nitrate (navy blue), sodium chloride (red), sodium borate (blue), sodium phosphate (magenta) and sodium citrate (olive). (**c**) Ice growth rates for the salted reference solutions with a zero ice volume fraction regime.

First, a series of control experiments were performed to examine the impact of the added salts on the dimensions and growth rates of ice crystals in 30 wt% sucrose solutions without AF(G)Ps. At the endpoint of the experiments, after an annealing time of 90 min, Shapiro–Wilk tests identified log-normal distributions of ice crystal radii ranging in size between 0.8 and 14.4 µm for all salted solutions without proteins (Figure 1a). The number average radius <*R*> was smallest for sodium phosphate (<*R*> = 8.0 µm) and largest for sodium nitrate (<*R*> = 8.4 µm). Similarly, ice recrystallization rates (*k*_d_ = ∂<R>^3^/∂t) were smallest for sodium phosphate (*k*_d_ = 7.1 µm^3^ min^−1^) and largest for sodium nitrate (*k*_d_ = 7.9 µm^3^ min^−1^). All five anions increased the ice recrystallization rate by roughly 30%, from ~5.5 µm^3^ min^−1^ for unsalted to 7.5 ± 0.3 µm^3^ min^−1^ for salted 30 wt% sucrose solutions (Figure 1b). As the addition of any solute to the control sucrose solution alters its freezing point and consequently changes its ice volume fraction [21], *φ*, we must rescale all ice crystal growth rates to an ice crystal volume fraction of zero, *φ*^0^, for an appropriate comparison (Figure 1c). As expected, after elimination of this influence of differences in ice crystal volume fractions on *k*_d_, we found that none of the anions had a statistically significant impact on the ice crystal growth rates (i.e., the added salts did not significantly modulate the diffusion of water molecules).

**Figure 2:**
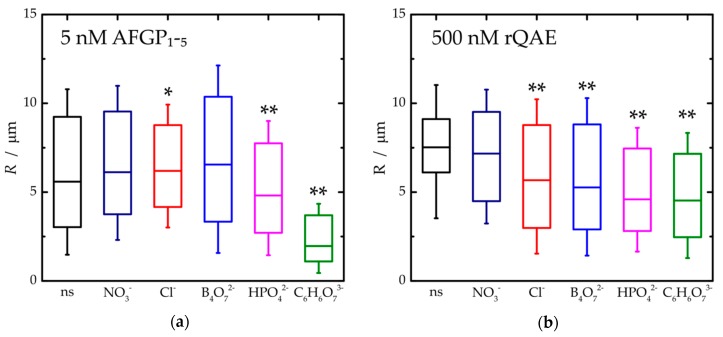
Boxplots of the ice crystal radii distributions after sample annealing at −7 °C for 90 min. (**a**) Samples containing 5 nM AFGP_1–5_ in 30 wt% sucrose (black), sodium nitrate (navy blue), sodium chloride (red), sodium borate (blue), sodium phosphate (magenta) and sodium citrate (olive). (**b**) Samples containing 500 nM QAE in 30 wt% sucrose (black), sodium nitrate (navy blue), sodium chloride (red), sodium borate (blue), sodium phosphate (magenta) and sodium citrate (olive). Median (line), minimum and maximum radii observed (whiskers). * *p*-value < 0.05, ** *p*-value < 0.01.

**Figure 3:**
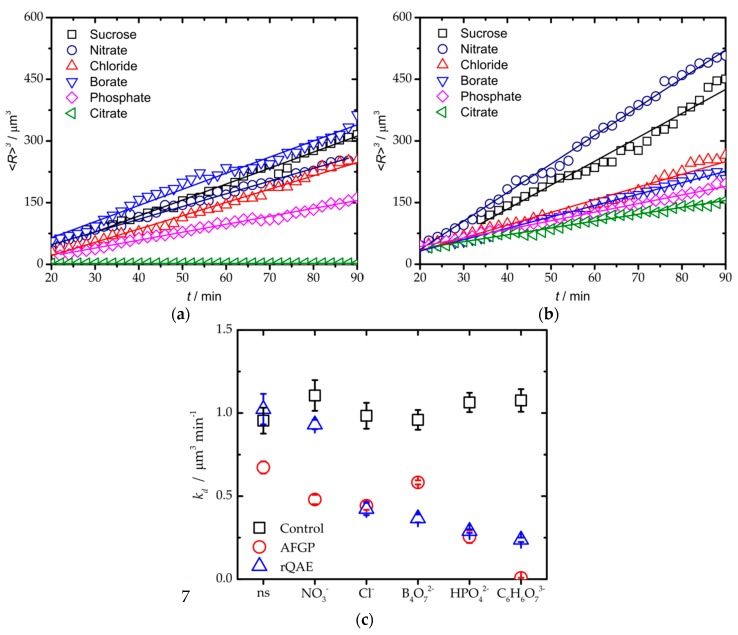
Ice growth rates (*k*_d_) after annealing at −7 °C for 90 min. (**a**) Samples containing 5 nM AFGP_1–5_ in 30 wt% sucrose (black) without added salts, and with (navy blue) sodium nitrate, (red) sodium chloride, (blue) sodium borate, (magenta) sodium phosphate and (olive) sodium citrate. (**b**) Samples containing 500 nM QAE in 30 wt% sucrose (black), sodium nitrate (navy blue), sodium chloride (red), sodium borate (blue), sodium phosphate (magenta) and sodium citrate (olive). (**c**) Ice growth rates for the salted reference solutions at a zero ice volume fraction regime for the reference solutions without proteins (black square), samples with 5 nM AFGP_1–5_ (red circle) and 500 nM rQAE (blue triangle).

Next, the impact of the salts on the IRI activity of AFGP_1–5_ was examined (Figure 2). The ice crystal distributions in all 5nM AFGP_1–5_ samples, both with and without added salts, were log-normal (Figures S4 and S5). Most distributions were also considerably skewed due to the presence of large amounts of small ice crystals. These remained for prolonged periods of time due to AF(G)P adsorption onto ice grains, which impeded both the melting of small ice crystals and the growth of large ice crystals [20,21]. Consequentially, the median was found below the average mean radius for these AFGP_1–5_ samples. As AFGPs are potent ice recrystallization inhibitors, <*R*> for the ice crystals remained considerably smaller (between 1.4 and 6.3 µm) in the salted sucrose solutions with AFGP_1–5_ (Figure 2a) for all salts except sodium borate (<*R*> ~6.9 µm). In accordance with LSW theory, we observed a linear increase in <*R*>^3^ as a function of time for all 5 nM AFGP_1–5_ samples (Figure 3a). The sodium salts significantly impacted the potency of AFGP_1–5_ to inhibit ice recrystallization as *k*_d_ rescaled to *φ*^0^ varied significantly amongst the salted sucrose solutions at a fixed AFGP_1–5_ concentration. Ranking AFGP_1–5_ on the order of ascending IRI potency from the least to the most active, we found B_4_O_7_^2−^ < NO_3_^−^ < Cl^−^ < HPO_4_^2−^ < C_6_H_6_O_7_^3−^ (Figure 3c). This trend in IRI activity followed the Hofmeister series for anions with the exception of sodium borate (*k*_d_ = 3.9 µm^3^ min^−1^). In fact, AFGP_1–5_ showed the same ice growth rate in the presence of sodium borate as without any salt in the solution. The complete loss of TH activity of AFGPs upon addition of borate ions is well-documented. It is caused by the cross-linking of AFGPs through esterification of the glycosylated threonine amino acids, which are crucial for both TH and IRI activity [23,24]. As borate can react with both AFGPs and sucrose, all borate samples were prepared by dissolution of the proteins in the borate solutions prior to the addition of sucrose. Remarkably, the addition of a six-fold molar excess of borate ions to AFGPs did not eliminate IRI activity completely. If the ability to inhibit ice recrystallization were completely suppressed, the *k*_d_ values for sodium borate containing AFGP_1–5_ samples should have been similar to those of the borate control solution (~7.2 µm^3^ min^−1^). We attribute this differential impact of borate on TH and IRI activity to a borate concentration-dependent decrease in the adsorption rate of AFGPs to ice. Indeed, an earlier study demonstrated that AFGPs exposed to 0.3 M of borate still adsorbed to ice seeds, albeit with three times less affinity, while TH activity was completely lost [25]. As ice-binding must be very strong for TH activity and less so for IRI activity, AFGPs exposed to low borate concentrations may be TH-defective yet IRI-active.

To examine whether the IRI activity of non-glycosylated antifreezes also follows the Hofmeister series for anions, we performed the same assays on rQAE at a concentration of 500 nM. This concentration was well suited for our purpose, as it is slightly below the inhibitory concentration for non-salted rQAE in 30 wt% sucrose, and thus a protein concentration at which additives that impact the IRI activity of rQAE would impact *k*_d_. The ice crystal radii distributions of most salted rQAE samples (with the exception of sodium nitrate) skewed towards smaller ice crystal sizes than the reference rQAE sample without added salt (Figure 2b). The mean dimensions of ice crystals at the endpoint of the rQAE IRI assay at 500nM (1.1 <*R*> 11.4 µm) were comparable to those of the AFGP_1–5_ IRI assay at a 100-fold lower concentration (0.7 <*R*> 10.7 µm). Ranking IRI activity for rQAE from weak to high based on *k*_d_ at *φ*^0^, we found NO_3_^−^ < Cl^−^ < B_4_O_7_^2−^ < HPO_4_^2−^ < C_6_H_6_O_7_^3−^ (Figure 3c). Once again, this trend is in line with the Hofmeister series for anions (this time without exceptions). Apparently, the reduction in borate concentration due to complexation of borate with sucrose was small, as its impact on ice recrystallization by rQAE was significant.

Interestingly, our quantitative analysis of the ice crystal growth rates when rescaled to vanishingly small ice crystal volume fractions demonstrated that the IRI activity of both rQAE and AFGP_1–5_ could be significantly enhanced by the addition of 100 mM sodium salts. Importantly, we arrived at the same conclusion and ranking of rQAE and AFGP_1–5_ in order of ascending potency if we used, as a quantitative metric of potency, the dimensionless parameter *r* = <*R*>_90_/<*R*>_20_, which corresponds to the ratio of the number average mean ice crystal radius at the endpoint of the IRI assay <*R*>_90_ to its equivalent at the start of the assay <*R*>_20_ (Table S1). The salt-induced enhancement of IRI activity followed the Hofmeister series for anions for both proteins with the exception of sodium borate, as it reacted with the hydroxyl groups of the threonine-linked disaccharides on AFGP_1–5_, thus impairing the ice-binding properties of the protein. Differences in salt composition (both type and concentration) of cryopreservation media can thus significantly impact the inhibition of ice recrystallization by AF(G)Ps, and may (partially) explain the apparently conflicting results in the scientific literature on the moderate success of AF(G)Ps as cryoprotectants. We attribute these salt effects to the same mechanisms put forward to rationalize the ‘salting-out’ of proteins, namely a salt-induced reduction in solubility due to specific interactions between salt ions and surface-accessible residues. For the antifreeze (glyco)proteins studied here, this effect may be augmented due to a salt gradient in the water–ice interface, which would further favor AF(G)P adsorption onto the ice crystals. Alternatively, specific ion–protein interactions could stabilize structural elements critical to the binding of AF(G)Ps to their respective ice crystal planes.

Importantly, our findings also reveal a new strategy to boost the performance of AFGPs as cryoprotectants. In all cases except for AFGP_1–5_ in the presence of sodium borate, sodium salts boosted both IRI and TH in accordance with the Hofmeister series for anions. By contrast, sodium borate raised the IRI activity of AFGP_1–5_, while it simultaneously diminished its thermal hysteresis (TH) activity [23]. This is exciting, as AFGPs are the most potent IRI active compounds known to date, but their utility in cryopreservation has remained limited. This is because their presence not only inhibits recrystallization, but also induces the formation of ice crystals with sharp edges, which are thought to cause cellular injury through, for example, membrane rupture.

## 4. Conclusions

In conclusion, we have quantified the impact of sodium salts at a fixed ionic strength of 100 mM on the ice recrystallization inhibition activity of two types of antifreeze (glyco)proteins. We found that all salts enhanced the IRI activity of rQAE and AFGP_1–5_ in accordance with the Hofmeister series for anions, with one exception, as previously reported by others for the TH activity of the insect antifreeze protein *Ri*AFP and AFGP_1–5_ [17,26]. Sodium nitrate had the weakest effect and sodium citrate the strongest. Surprisingly, sodium borate retained IRI activity of AFGP_1–5_ despite its reaction with the hydroxyl functionalities of AFGP_1–5_, which is known to diminish TH activity. The presence of salts clearly affected the performance of AF(G)Ps, which may find use as additives in cryopreservation media to inhibit ice recrystallization and thereby alleviate cryoinjury. Our findings contribute to an improved understanding of the physical mechanisms that underpin the differential impact of excipients, specifically salts, on the performance of cryopreservation formulations.

## Supplementary Materials

The following material is available online at https://www.mdpi.com/2218-273X/9/8/347/s1, Figure S1: Data acquisition and ice crystal analysis. Figure S2: Determination of ice growth rates from samples. Figure S3: sSart and end-point microphotographs. Figure S4: Log-normal ice crystal radii distributions of AFGP_1–5_. Figure S5: Log-nominal ice crystal radii distributions of rQAE. Table S1: Statistical analysis of normality. Table S2: Mann–Whitney–Wilcoxon tests.
