# Supplementary material for: The Impact of Salts on the Ice Recrystallization Inhibition Activity of Antifreeze (Glyco)Proteins
Source: Biomolecules. 2019 Aug 6;9(8):347. doi: 10.3390/biom9080347 (PMC6724029; doi:10.3390/biom9080347)
Supplement: Supplementary file 1 [file biomolecules-09-00347-s001.pdf]

SUPPORTING INFORMATION

The Impact of Salts on the Ice Recrystallization Inhibition Activity of Antifreeze (Glyco)Proteins

Romà Surís-Valls, Ilja K. Voets*

Laboratory of Self-Organizing Soft Matter, Laboratory of Macro-Organic Chemistry, Department of Chemical Engineering and Chemistry & Institute for Complex Molecular Systems, Eindhoven University of Technology, Post Office Box 513, 5600 MD Eindhoven, The Netherlands

* Correspondence: i.voets@tue.nl; Tel.: +31-40-247-5303

Analysis of microphotographs from the IRI assay

We apply a mask, followed by conversion to binary image and inversion, on the microphotographs obtained in the IRI assay using ImageJ (**Figure S1**). We determine the area for each of the crystals in the image by using the built-in particle analysis. We use a Matlab script to determine the radii of an equivalent circle from each of the areas and then we produce a size distribution. The cubic number average radius, $\langle R \rangle^3$, increases over time, following the theory of Lifshitz, Slyozov and Wagner [1]. The growth rate constant, $k_{\mathrm{d}}(c)$, is determined from the slope of the linear increase of $\langle R \rangle^3$in time (**Figure S2**).

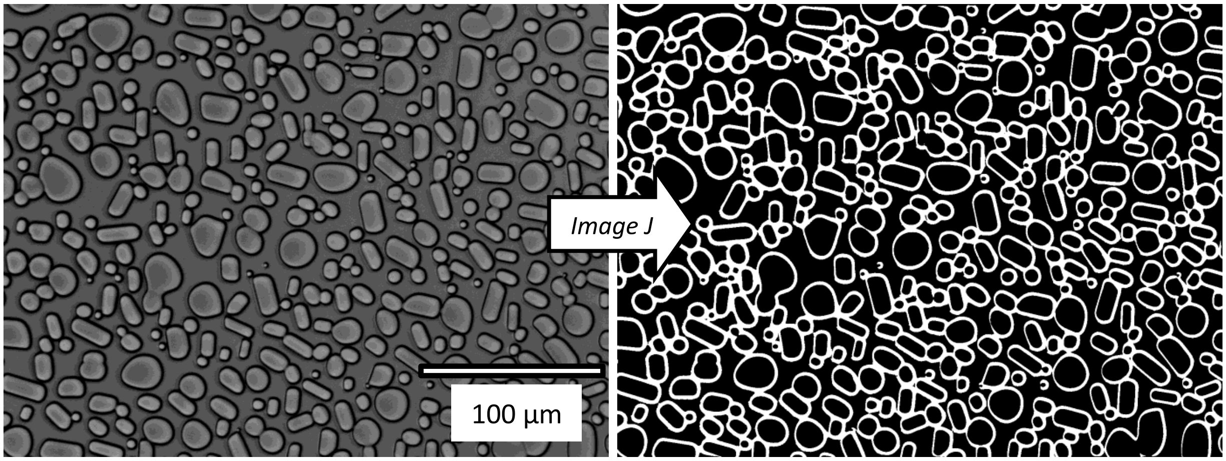

Figure S1. The microphotograph of sucrose solution 30% after 50 minutes of annealing at -7 °C (left) is converted into a binary image using ImageJ (right) to determine the radii of the ice crystals in the sample.

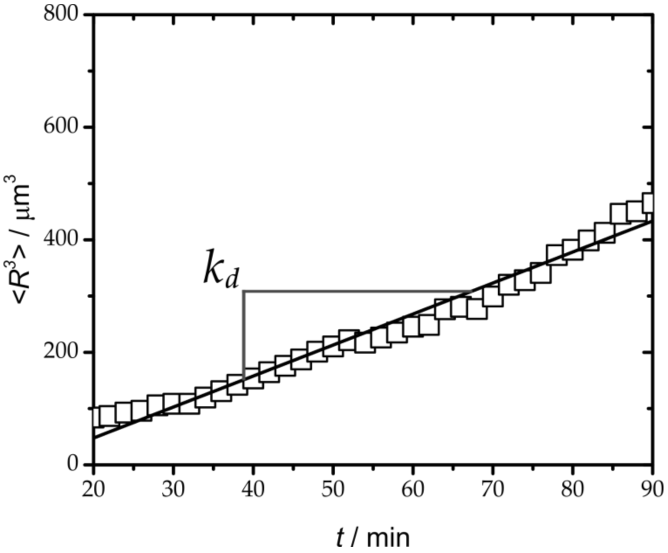

Figure S2. The cubic number average radius of the ice crystals increases linearly in time and the slope gives the growth rate constant k_d.

Microphotographs of AF(G)P solutions

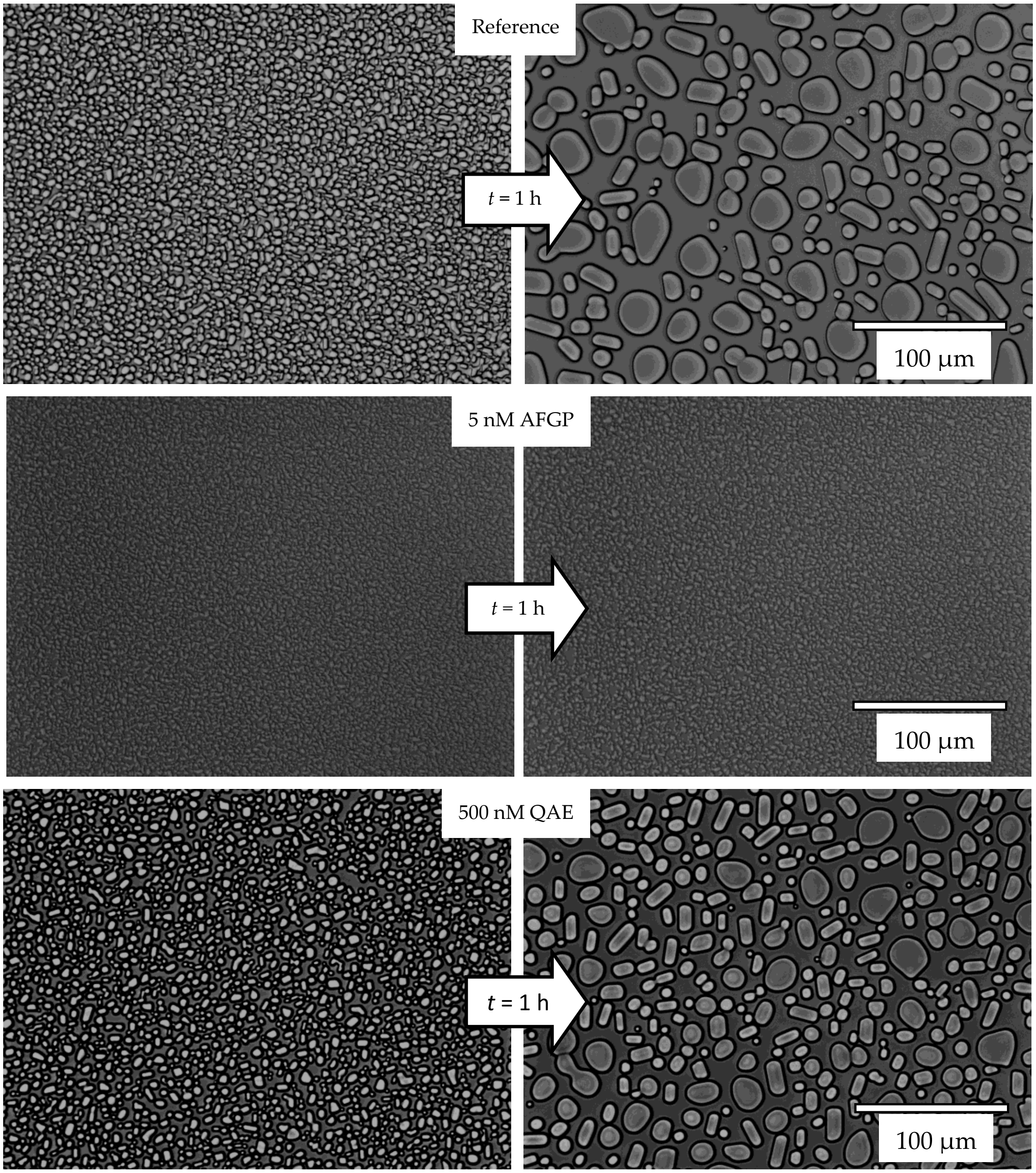

Figure S3. Microphotographs of the sodium citrate salted solution (+ 30 wt% sucrose) at -7 °C at the start (left) and of the one hour of annealing (right).

Ice crystal distributions of salted solutions containing AF(G)Ps

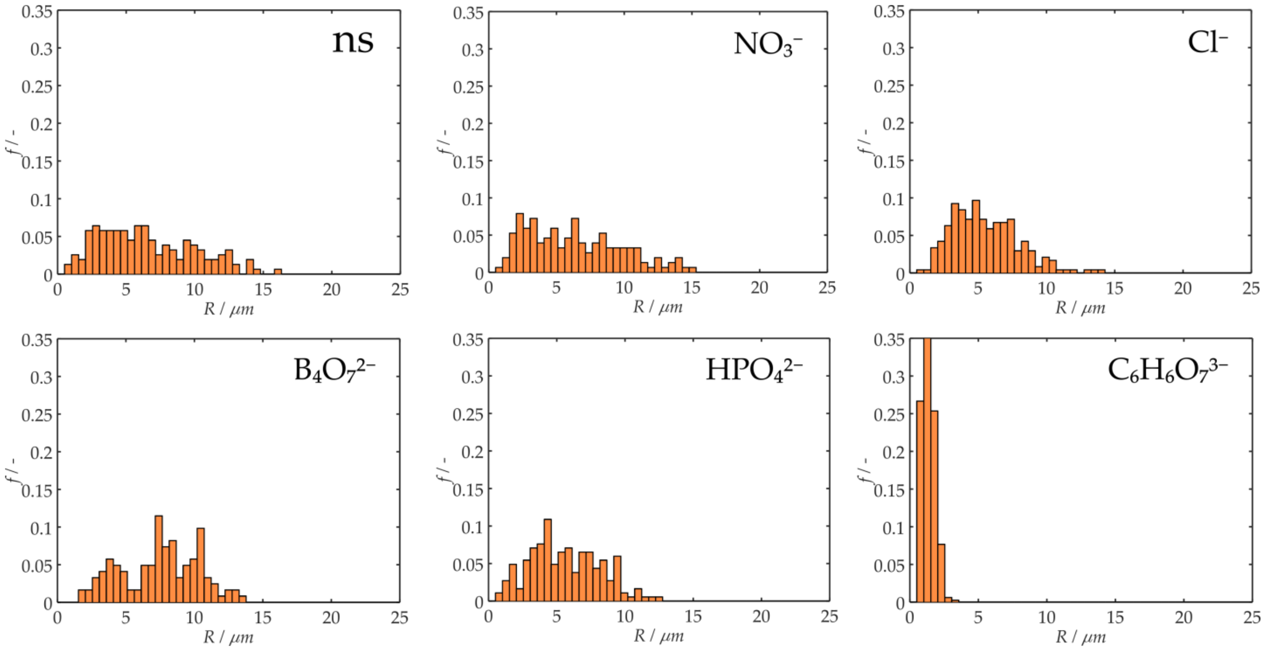

Figure S4. Ice crystal radii distributions at the end point of experiments for samples of AFGP1-5 in presence of different anions.

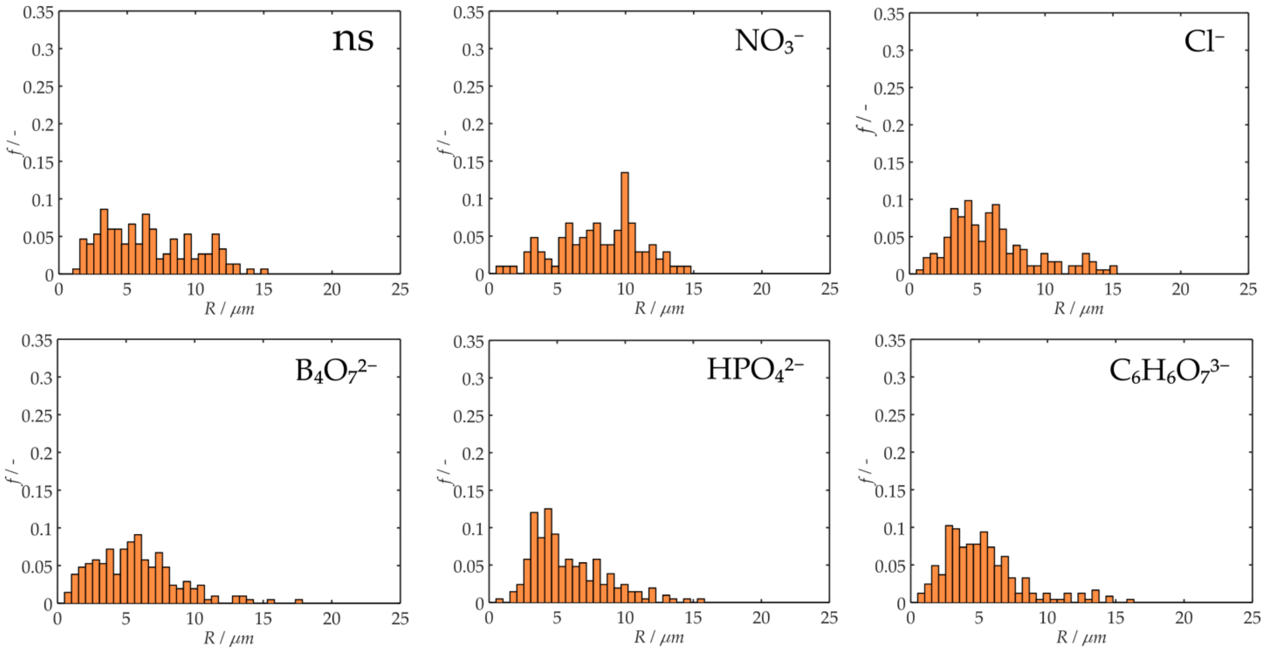

Figure S5. Ice crystal radii distributions at the end point of experiments for samples of rQAE in presence of different anions.

Rescaling ice growth rates to zero ice volume fraction

The addition of any solute to the control sucrose solution alters its freezing point and consequently changes its ice volume fraction. To properly compare samples with different amounts of solutes, rescaling all ice crystal growth rates to an ice crystal volume fraction of zero is necessary. We used Formula(1) to rescale the ice growth rate to zero volume fraction

$$k_d(\varphi) = k_{do} \frac{\alpha^3}{1-p\varphi^{1/3}} \quad (1)$$

According to literature, the α can be adjusted to 1 since its dependence to the ice volume fraction is negligible. The parameter p is a scaling factor that is calculated from the dependence from ice growth rates, ice volume fraction and annealing temperature and can be approximated to 1.32 for annealing temperatures of -7 ^{o}C [1].

To provide ice growth rates that can be compared between different conditions and experimental protocols, the rates from the samples (k_s) to compare must be normalized by the reference control (30 wt% sucrose) and scaled to $\phi = 0$ using Formula (2).

$$k_{so} = \frac{k_{do} k_s \varphi_s}{k_d \varphi_d} \quad (2)$$

Analysis of ice crystal size distributions

The distributions of ice crystals for solutions of $AFGP_{1-5}$ and QAE in nitrate, chloride, borate, phosphate and citrate buffers are tested for normality using a Shapiro Wilks test. Normality is rejected for p-values below the 0.05 level (**Table S1**).

Table S1. Statistics analysis and result of normality test (Shapiro-Wilks)

	**AFGP$_{1-5}$**			**rQAE**		
**Distribution**	**median**	**st.dev.**	**p-value**	**median**	**st.dev.**	**p-value**
Reference	5.59	3.1		7.76	1.8	
Nitrate	6.12	2.9	**0.055**	7.17	1.7	**0.023**
Chloride	6.20	2.3	**0.530**	5.67	1.7	**< 0.001**
Borate	6.55	3.5	**< 0.001**	4.27	1.7	**< 0.001**
Phosphate	4.81	2.5	**< 0.001**	4.59	1.7	**< 0.001**
Citrate	1.96	1.5	**< 0.001**	4.53	1.7	**< 0.001**

Since almost all the distributions are not normally distribution, the non-parametric Mann Whitney Wilcoxon test is performed to compare the median value of ice crystal size to look for statistical differences. The test is implemented between the reference and the different buffers to check for statistical differences to confirm the enhancement effect produced by salt addition (Table S2). The result shows that only the medians for nitrate are equal to the reference for both proteins, whereas borate affects the ice recrystallization in the case of QAE, but not for $AFGP_{1-5}$.

Table S2. Parameters and results for non-parametric Mann Whitney Wilcoxon test

	$AFGP_{1-5}$			rQAE		
**Test pair**	**p-value**	**Signif. level**	**Reject equal medians**	**p-value**	**Signif. level**	**Reject equal medians**
Control – nitrate	**0.069**	0.05	No	**0.055**	0.05	No
Control – chloride	**0.042**	0.05	Yes	**< 0.001**	0.05	Yes
Control – borate	**0.081**	0.05	No	**< 0.001**	0.05	Yes
Control – phosphate	**0.007**	0.05	Yes	**< 0.001**	0.05	Yes
Control – citrate	**< 0.001**	0.05	Yes	**< 0.001**	0.05	Yes

Table S3. Number average radius of ice crystals at the start point ($\langle R\rangle_{20}$), end point ($\langle R\rangle_{90}$) of the experiments and the dimensionless parameter *r*

	**Control**			$AFGP_{1-5}$			**rQAE**		
	$\langle R\rangle_{90}$	$\langle R\rangle_{20}$	*r*	$\langle R\rangle_{90}$	$\langle R\rangle_{20}$	*r*	$\langle R\rangle_{90}$	$\langle R\rangle_{20}$	*r*
no salts	7.4	4.6	1.61	6.7	4.1	1.63	7.7	4.7	1.64
NO_3^-	8.4	5.6	1.50	6.3	4.1	1.54	7.9	5.0	1.58
Cl^-	8.3	5.4	1.54	6.3	4.3	1.47	6.4	4.2	1.52
$B_4O_7^{2-}$	8.1	5.4	1.50	6.9	4.3	1.60	5.9	4.0	1.48
HPO_4^{2-}	8.0	5.2	1.54	5.4	3.8	1.42	5.7	4.2	1.36
$C_6H_6O_7^{3-}$	8.2	5.3	1.55	1.4	1.3	1.08	5.3	4.0	1.34

References

1. Budke, C.; Heggemann, C.; Koch, M.; Sewald, N.; Koop, T. Ice recrystallization kinetics in the presence of synthetic antifreeze glycoprotein analogues using the framework of LSW theory. *J Phys Chem B* **2009**, *113*, 2865-2873, doi:https://doi.org/10.1021/jp805726e.
